# Rigorous Training of Dogs Leads to High Accuracy in Human Scent Matching-To-Sample Performance

**DOI:** 10.1371/journal.pone.0146963

**Published:** 2016-02-10

**Authors:** Sophie Marchal, Olivier Bregeras, Didier Puaux, Rémi Gervais, Barbara Ferry

**Affiliations:** 1 Division of the Technical and Scientific Police (DTSP), Central Direction of the Judicial Police, Central Service of the Judicial Identity, Forensic Section, 31 Avenue Franklin Roosevelt, F-69134, Ecully, Cedex, France; 2 Centre de Recherche en Neurosciences de Lyon— UMR CNRS 5292 — INSERM U 1028 — Université Claude Bernard Lyon 1, Université de Lyon, 50 avenue Tony Garnier, F-69366, Lyon, France; Université de Technologie de Compiègne, FRANCE

## Abstract

Human scent identification is based on a matching-to-sample task in which trained dogs are required to compare a scent sample collected from an object found at a crime scene to that of a suspect. Based on dogs’ greater olfactory ability to detect and process odours, this method has been used in forensic investigations to identify the odour of a suspect at a crime scene. The excellent reliability and reproducibility of the method largely depend on rigor in dog training. The present study describes the various steps of training that lead to high sensitivity scores, with dogs matching samples with 90% efficiency when the complexity of the scents presented during the task in the sample is similar to that presented in the in lineups, and specificity reaching a ceiling, with no false alarms in human scent matching-to-sample tasks. This high level of accuracy ensures reliable results in judicial human scent identification tests. Also, our data should convince law enforcement authorities to use these results as official forensic evidence when dogs are trained appropriately.

## Introduction

Olfactory cues provide information about food, mates, offspring, predators, prey and pathogens [1, 2]. Because detection of these compounds is essential for survival, the majority of animals have developed a highly sophisticated olfactory system during the course of evolution [3], with thousands of volatile compounds perceived as distinct odours [1].

Among species that show remarkable olfactory detection ability, dogs have long been used in a variety of forensic areas [4–8]. In particular, the ability of dogs to identify and discriminate between human odours has long been documented [9] and numerous experimental studies have shown that dogs are able to detect, identify and memorize the odour of a particular person with high specificity.

Gas chromatography-mass spectrometry studies showed that each human scent consists of a combination of volatile components produced from the skin [10, 11] and differing in ratio from person to person, along with some compounds that are unique to certain individuals. This combination, which has been shown to be constant and reproducible over time [12–15], contributes to the individuality and uniqueness of human scent [10, 16–18]. This finding likewise includes identical twins’ individual scents [19, 20].

In addition, studies have shown that human scent can persist and maintain its main chemical features for a significant period of time in a particular place or/and on an object that was manipulated by a subject [12–15].

Exploiting the fact that a person’s odour is left at every location, object or pathway with which the person has come into contact [21], the uniqueness and persistence of human scent have been used forensically, in line-ups, to identify particular human scents found at crime scenes. Developped early by Schoon and De Bruin (1994) [22] and refined since then [23, 24], the human scent identification line-up is a task in which trained dogs are presented with scent collected from a crime scene (“evidence” scent) and are required to compare this sample to a selection of human scents (“comparison” scents, usually 5 or 6 in number) collected from persons not involved into the crime but also from a possible suspect (“target” scent). If the target scent of the suspect in the line-up matches the evidence scent, the dog shows a typical conditioned response (usually, sitting or lying down) at the target station. The human scent line-up method, which has been used with dogs since the beginning of the 20th century (see [25] for review), relies on a classical matching-to-sample procedure, in which the dog’s conditioned response to a correspondence between target scent and sample is reinforced by food, whereas responses to any non-identical comparisons are not reinforced [26].

Despite the great ability shown by dogs in line-up tasks [23, 24] the method has not gained widespread recognition in the worldwide forensic community, and human scent identification results remain a controversial form of legal evidence [27].

One reason for reluctance to use this method seems to be a lack of international standards for the way in which dogs are trained, certified and used [28]. In this vein, studies showed that the accuracy of human scent line-up identification results depends directly on the quality of the dog’s training [19, 20, 29–32]. The present study therefore aimed to provide a precise description of the various steps of the training which is routinely used in the French Division of the Technical and Scientific Police (DTSP, Ecully, France) and which lead to a very high success rates in human olfactory matching-to-sample tasks.

## Material and Methods

### Animals

Data obtained for 13 Shepherd dogs (German: 1 female, 8 males; Belgian: 1 female, 3 males) over a period of 10 and a half years were used in this study.

All of the dogs came from breeding establishments in Hungary (Dunakeszy School of Dog Handlers, Dunakeszy, Hungary) and France (Dogs Company, Mounsempe, France; and La Petite Renardière, Saint-Romain de Jallonias, France), where they were raised and selected for their olfactory abilities. Each dog had its own living box (2m wide × 1.9m high × 4m long) with an elevated straw-bottomed wooden crate (0.9m wide × 0.9m high × 1.5m long). Dogs had access to two recreation areas (63.5m^2^), with water basins and various games, for about 3 hours every day, alone or in pairs.

The dogs were trained 5 days per week when possible (from monday to friday), and rested during the weekend. The dogs were aged between 10 months to 3 years at the beginning of the training.

### Food

The animals were fed once a day between 4:00 and 6:00 PM, with balanced diet (Royal Canin, 19.5 g/kg) and access to water ad libitum.

### Work room

The dogs were trained in a temperature-controlled (16–24°C) rubber-floored room. Five jars on metal tripods were lined up along a dark blue rubber ground line (0.2m × 9m). The work room floor was washed every morning with clean water without detergent and once a week with an automated cleaning appliance.

### Scent collection

All human scents were collected by a qualified technician, wearing a special sterile paper suit and powder-free nitrile examination gloves. Scents were collected and stored according to a precise procedure routinely used by French scientific police officers (CS018 and CS033 from the Resources and Management Services of the National Police, DRCPN). In the case the suspect refuses to agree with scent collection, he or she can be condemned to a prison sentence according to a precise procedure laid down in Article 55.1 of the French Criminal Procedure Code (http://www.legifrance.gouv.fr/). This enables a police officer to collect any scent traces from the clothes or personal objects belonging to the suspect put in custody, to support the investigation.

#### Body scent (BS) collection

For control hand scent samples, subjects were asked to hold and manipulate 2 Kapp Péterné^®^ (Hungary) cotton squares in each hand simultaneously for 10 min. At the end of the collection time, the technician placed the cotton squares in a sterile glass jar (Verretech T082, 750 ml) with sterile clamps. The technician then closed the lid, and all the glass jars containing cotton squares were labeled with codes and specific information, including date, subject’s identity and gender, smoker/non-smoker status, exact times of start and end of scent collection, and the identity of the technician. The scent of a suspect held in custody was collected similarly, after obtaining their consent. The cotton squares were collected, placed in a jar labeled as previously described and, when the lid was closed, the jar was sealed and stored in a specific room.

#### Trace Scent (TS) collection

Olfactory traces of control subjects, suspects and crime-scene objects were collected similarly. For this, a technician placed 1 to 5 Kapp Péterné^®^ cotton squares directly in contact with the object or clothes, using sterile clamps. Then, the object or clothes with the cotton squares were wrapped in aluminum foil for at least 1 hour. At the end of that time, the technician removed the aluminum foil, using sterile clamps, and the cotton squares were all placed in the same jar, labeled with codes and specific information, including date, subject’s identity and gender, smoker/non-smoker status, exact times of start and end of scent collection, type of object, type of material, precise location of the object and the identity of the technician. Then the lid of the jar was closed; only jars with TS collected from suspects and from crime-scene were sealed. Jars containing control and suspect TSs were stored in distinct rooms, at a temperature between 15°C and 25°C under constant humidity. Scents can be kept for 10 years or even more. Samples were kept in the jar for at least 24 hours before being used in line-up tests.

### Experimental design of dog training

The training consisted in the dog acquiring the human olfactory matching-to-sample task, in which successful choice of the odour matching the sample is followed by reinforcement (food or a dog treat). The training program took approximately 18–20 months, comprising initial training (steps 1 to 5, each step lasting approximately 2 months) and continuous training (8–10 months and throughout the dog’s life). At the end of the training, dogs entered the judicial case program. Each daily training session included a serie of 6–8 line-up trials, with each correct response rewarded by food (10 g Knacki^®^ sausage) or a game (a ball given at the end of trial); each type of reward being chosen by the dog handler relatively to the sensibility of their dogs. The number of daily line-ups was adjusted very early during the procedure validation by the number of trials beyond which the dog handler detected any change in their behavior indicating a decrease in the dog’s motivation and attention. The dog experimentation was conducted as a part of routine training.

#### Initial training

During the various steps, jars without lids were lined up in the work room by a qualified technician wearing a special sterile paper suit and powder-free nitrile examination gloves.

Steps 1 to 3: acquisition of learning rules (sniffing behaviour and the lying down conditioned response).

**Fig 1:**
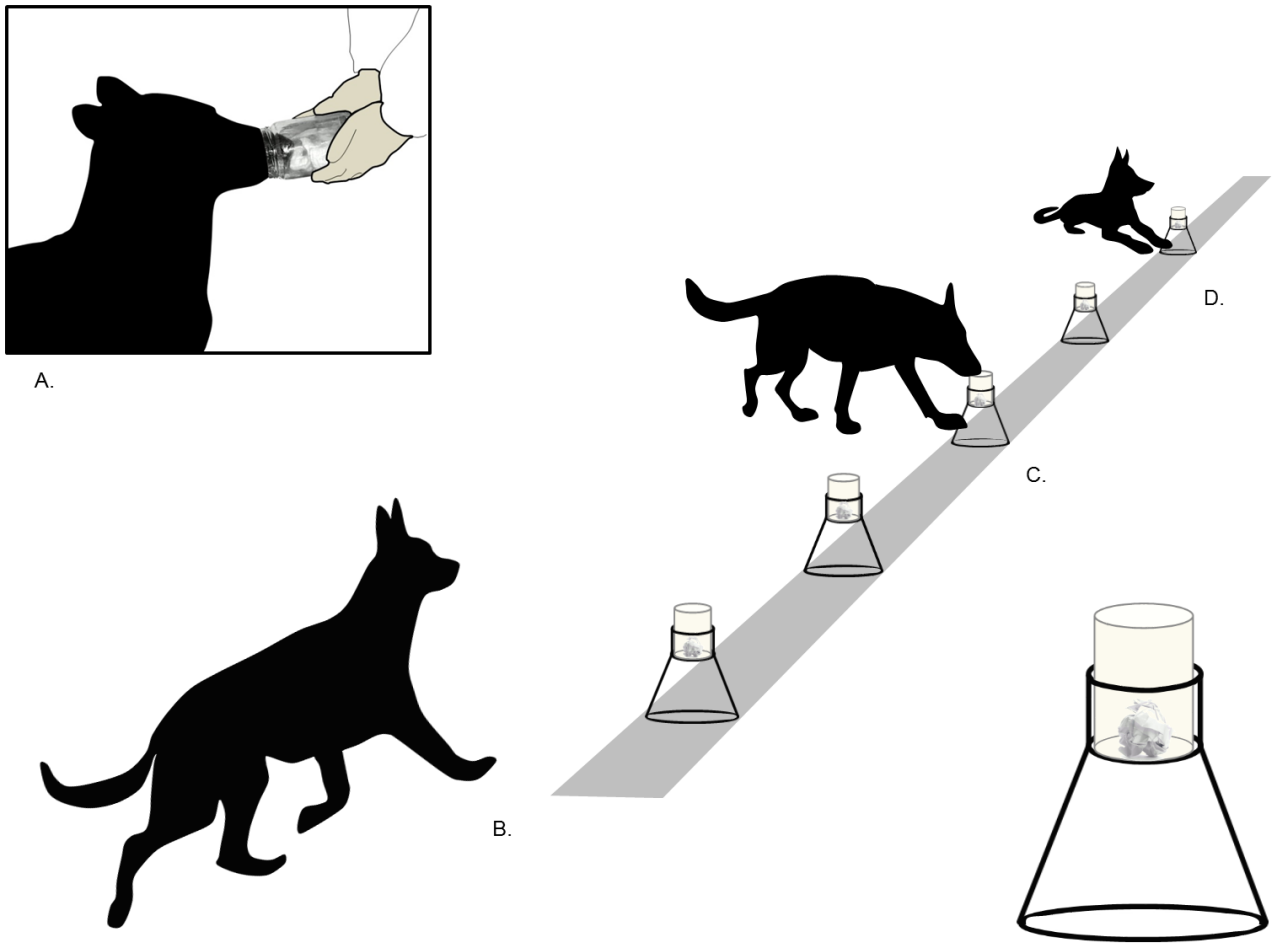
Four positions of a trained dog during the human scent line-up task. 1A: the dog sampled the target scent inside the jar. 1B: the dog was then required to walk the line from the starting point. 1C: the dog sniffed in all of the jars along the line. 1D: when an odour in the line matched the target sample, the dog lay down in front of the corresponding jar. An enlargement of the drawing representing the tripod we use in our procedure is represented at the bottom of the figure.

Each session started when the dog handler, wearing powder-free nitrile examination gloves, presented an open jar containing a clean cotton square (Kapp Péterné^®^) together with the reward at a starting point and encouraged the dog to sniff inside for a minimum of 5 sec (Fig 1A). At the end of the 5 sec, the dog handler gently guided the dog, by its leash, backward and forward over the line-up and encouraged it to stop and sniff inside each jar by standing beside the dog in front of it. The dogs were trained at least once a day. The procedure was repeated again until the daily line-up trials were completed. The total number of line-ups performed by the dogs and the position of the target jar were recorded in the report. In step 1, all the jars in the line-up contained a piece of sausage and a clean cotton square; dogs were rewarded when they placed their nose in all jars. In step 2, all jars contained a clean cotton square but only 2 (randomly placed) contained the reinforcement; dogs were rewarded when they placed their nose in these 2 jars only. In step 3, 1 jar only (randomly placed) contained the reinforcement, together with a clean cotton square; dogs were rewarded when they placed their nose in this jar and lay down in front of it. The experimental group comprised 5 dogs (*Frost*, *Diva*, *Cisko*, *Bac* and *Athos*). When the dogs showed 100% correct response (lying down in the front of the rewarded jar) over 16 trials, they entered step 4. The mean total number of trials to reach this criterion in step 3 was 156 ± 24 and corresponded to 3 to 4 weeks of training. The mean total number of trials for each dog needed to achieve the three steps was 363 ± 25 and corresponded to 10 to 11 weeks of training.

Step 4: Human scent recognition task

Two dogs (*Cartmen* and *Batu*) that had been pre-trained in Hungary with a procedure similar to ours, arrived at the French DTSP facilities (Ecully, France) and were included in the experimental group. During this step, 1 jar containing a human odour (target scent, corresponding to a cotton square with human BS collected 24 hours previously from individuals belonging to the police service) was placed, without a lid, in the line-up by a qualified technician, with 4 other jars containing clean cotton squares. Handlers were blind to the target scent’s position in the line-up. Each session started when the dog was taken to the work room, where the handler removed the leash and presented the open jar containing the target sample (also included in the line-up as the target scent) at the starting point for a minimum of 5 sec. Then the dog was allowed to search alone along the line-up for the matching scent (Fig 1B and 1C). While the dog was walking the line, the handler stood next to the technician, looking in the opposite direction, in order to avoid any possible influence (e.g. by his attitude or through a visual communication) on the dog performance. When the dog stopped and lay beside the jar containing the matching odour, the technician indicated it to the handler, who rewarded the dog at the correct jar station (i.e., reinforcement was contingent upon the lying down response). This behaviour was noted as a Hit response (Fig 1B). Sometimes the dog did not stop in front of the jar containing the target scent. This behaviour was noted as a Miss response.

For each dog, the daily training session included a serie of 6–8 line-up trials and scents from different subjects (sample and target) were used every day. The positions of the jars were randomly changed by the technician throughout the successive trials, but not the position of the metal tripod; thus, the dog’s choices could not be driven by any odour left on the tripod or the jar. Scents were changed between each dog's series of trials. The total number of line-ups performed by the dog and the position of the target were recorded in the report. The total number of trials with Hit and Miss for each session and for each dog was used to calculate accuracy score corresponding to Hits / total of trials. Animals that reached the criterion of 95% accuracy in correct responses for at least 20 successive trials entered the next step. The mean total number of trials to reach this criterion was 226 ± 30 and corresponded to 6 weeks of training. The experimental group comprised 7 dogs (*Frost*, *Diva*, *Cisko*, *Bac*, *Athos*, *Cartmen* and *Batu)*.

Step 5: human scent matching-to-sample task

Two dogs (*Dunak* and *Carlos*) that had been pre-trained in Hungary with a procedure similar to ours arrived at the French DTSP facilities (Ecully, France) and were included in the experimental group.

In this step, 1 jar contained the target scent (BS or TS) was randomly placed in the line-up with 4 other jars containing comparison scents (BS or TS collected from unrelated persons randomly chosen in the population but of the same status of sex, age and ethnicity). Handlers were blind to the target scent’s position in the line-up. Three matching combinations between sample and target were used during the procedure: BS in sample / BS in line-up (BS/BS), BS in sample / TS in line-up (BS/TS), and TS in sample/ BS in line-up (TS/BS). The scents presented in the line-up were systematically all of the same type (all BS or all TS). Each session started when the dog was taken to the work room and the handler removed the leash and presented the open jar containing the target sample to the dog at the starting point for 5 sec. Then the dog was allowed to search alone along the line-up for an odour matching the target sample (Fig 1B and 1C). While the dog was walking the line, the handler stood next to the technician, looking in the opposite direction. When the dog stopped and lay down beside the jar containing the matching odour (Hit) and ignored the distracters, the technician indicated it to the handler, who rewarded the dog at the correct jar station. When the dog stopped and lay down beside the wrong jar, the response was noted as a False Alarm (FA). When the dog walked on without stopping at the correct jar, the response was noted as a Miss. Probe tests were inserted during the training session (no target scent in the line-up) and, when the dog continued the line-up without pause, the response was noted as a Correct Rejection (CR) and a reward was delivered only at the end of the probe test when the dog came back to its handler, in order to avoid learning that CR responses to distracters could be reinforced. All responses (Hits FA, Misses and CR) were noted in the report.

For each dog, the daily training session included a serie of 6–8 line-up trials and scents from different subjects (sample and target) were used every day. The positions of the jars were randomly changed by the technician throughout the successive trials, but not the position of the metal tripod; thus, the dog’s choices could not be driven by any odour left on the tripod or the jar. Scents were changed between each dog's series of trials. The total number of line-ups performed by the dog and the position of the target were recorded in the report.

The total number of trials with Hit and Miss for each session and for each dog was used to calculate specificity score corresponding to CRs / (CRs + FAs).

Animals that reached the criterion of 100% specificity in correct responses for at least 100 successive trials (corresponding to 12 sessions or 2 to 3 weeks of training) entered the continuous training program. The mean total number of trials to reach this criterion was 377 ± 57 and corresponded to 9 to 10 weeks of training.

Only dogs that gave no False Alarms over 200 trials during step 5 (corresponding to the last 24 sessions or 4 to 5 weeks of training) entered the judicial case program. The experimental group comprised 9 dogs (*Frost*, *Diva*, *Cisko*, *Bac*, *Athos*, *Cartmen*, *Batu*, *Dunak* and *Carlos*).

#### Continuous training

Continuous training took place after step 5 of initial training, concomitantly to the judicial case program. Four dogs (*Tolatos*, *Vidra*, *Rexi* and *Yolan*), that had been pre-trained in Hungary with a procedure similar to ours, arrived at the French DTSP facilities (Ecully, France) and were included in the experimental group at this stage. The total number of dogs constituting the experimental group was thus 13. The procedure was similar to that described for step 5 of initial training except that 4 matching combinations between the target sample and the target scent presented in the line-up were used: BS/BS, BS/TS, TS/BS and TS/TS. Only dogs that gave no False Alarms over 200 trials during step 5 and continuous training entered the judicial case program. Continuous training continued between each judicial case procedure and throughout the working period of the dog’s life. One dog (Athos) was excluded from the group because its records were accidently lost. The total number of animals in the experimental group was then 12.

### Judicial cases (court cases)

#### Judicial cases identification task

About 15 minutes before the start of the judicial case task, human olfactory matching-to-sample performance was evaluated on a pre-case proficiency test. For this, dogs were tested on 3 line-ups of human odours with a procedure similar to continuous training. All dogs present in the French DTSP facilities (Ecully, France) since 2007 underwent this test, and those that showed 100% accuracy ([CRs + Hits] / total) in correct responses were enrolled in the judicial case identification task.

The purpose of the identification task was to make a match between a TS collected from a crime-scene object and the scent collected from a suspect or victim (BS or TS). The general procedure was similar to continuous training except that 2 matching combinations were used: TS/BS (79.8% of cases) and BS/TS (20.2% of cases). Probe tests were inserted between trials. Positive identification was noted in the official report when the dog lay down in front of the jar containing the matching odour. In that case, the line-up was repeated by the dog and the trial was recorded by a video camera. In case of a Miss, the trial was considered negative and the technician noted absence of identification. A Miss response meant that the dog did not match the sample with the target, but did not necessarily imply that the target scent was not present in the sample or that the suspect was not present at the crime scene. Hits and Misses were always confirmed with 100% consistency by the other dogs working on the same case (the same day or some days later). When the dogs completed all of the tests, the scent identification was officially validated and the report indicated whether an association had been made between the scent from the suspect and the collected evidence scent.

### Ethical approval

Ethical clearance to conduct this research project on dogs was obtained from the Dog Care and Use Committee of The Resource and Management Services of the National Police (DRCPN) in accordance with official procedures n°CC016 and CC017. The care and use of dogs in all experimental procedures met the requirements imposed by the international police (InterPol) legislation and were approved by the review board of the Central Direction of the French Judicial Police. All control scents were collected from volunteers according to the procedure previously described with their verbal and written consent. The human volunteer scent collection procedures complied with the Declaration of Helsinki for Medical Research involving Human Subjects and were approved by the review board of InterPol and the Central Direction of the French Judicial Police specifically designed to evaluate the potential ethical concerns of research on human subjects.

Procedures for trace and body scent collection from suspects in custody met all the requirements of Article 55.1 of the French Penal Procedure Code and were approved by the review board of the Central Direction of the French Judicial Police.

### Data analysis

All statistical analyses were performed with the SYSTAT 12.0^®^ program. The mean number of trials to criterion (± S.E.M.) in the 5 steps of initial training was analyzed on one-way ANOVA with post-hoc Bonferroni tests for intragroup comparison. In continuous training, sensitivity (true positive rate: hits / hits + misses and specificity (true negative rate: FAs / FAs + CRs) were analyzed on one-way ANOVAs with post-hoc Fisher tests. Differences in sensitivity between olfactory combinations (BS/BS, BS/TS, TS/TS and TS/BS) were analyzed on two-way repeated-measures ANOVA with Type of Combination as the between-subjects factor and Period as the within-subjects factor. Pairwise intergroup and intragroup comparisons were performed on one-way ANOVAs with post-hoc Fisher tests. For all statistical comparisons, the significance threshold was set at 0.05. The number of animals per group is indicated in the figure legends.

## Results

### Initial training

**Fig 2:**
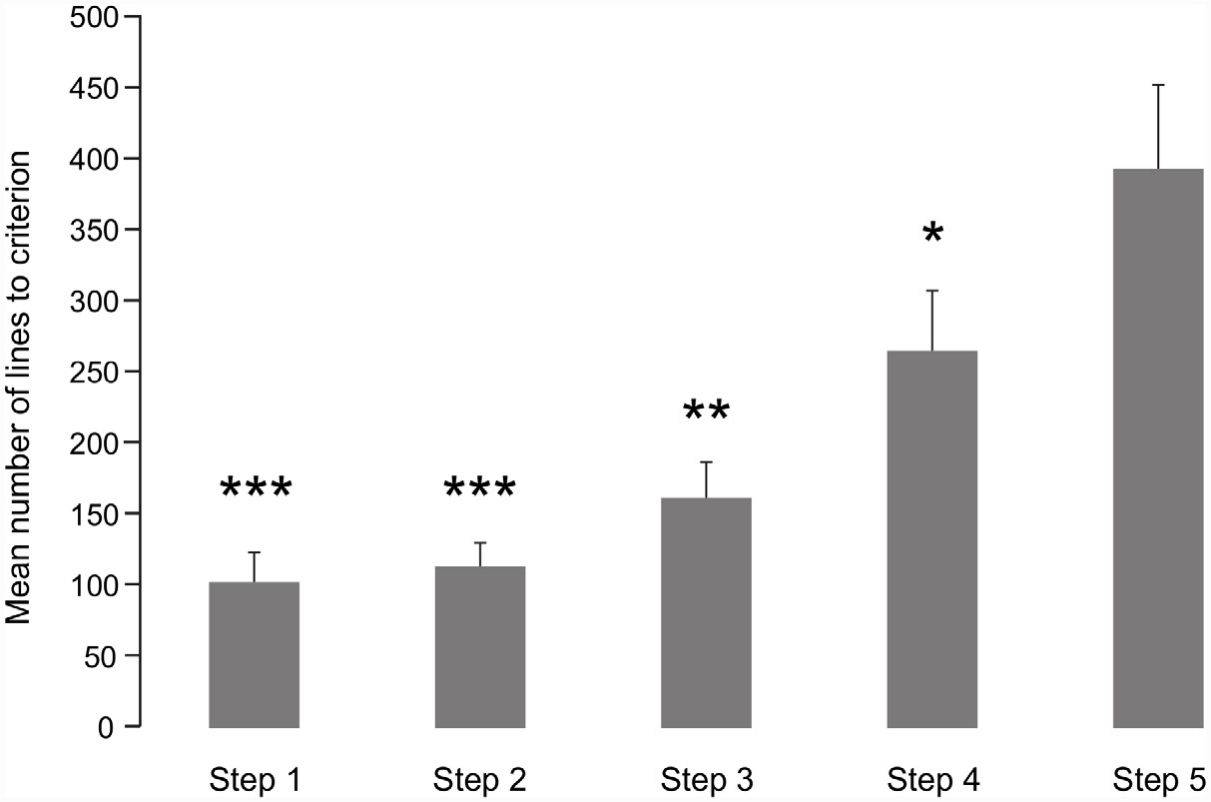
Mean number of trials (± S.E.M.) to reach criterion at each step of initial training. Number of dogs in the experimental groups: n = 5 in steps 1 to 3; n = 7 in step 4 and n = 9 in step 5. ***, ** and *: *P* < 0.001, 0.01 and 0.05 respectively, compared to step 5, with post-hoc Bonferroni tests.

As shown in Fig 2 (S1 Data), the mean number of trials required to reach criterion in the various steps of initial training in the experimental group varied depending on the type of task and significantly increased over each successive step (one-way ANOVA on factor Task (F(4,26) = 10.18; *P* < 0.001): i.e., the number of trials increased with the complexity of the task. The specificity scores at the end of step 5 demonstrated that dogs fully acquired the human olfactory matching-to-sample task with our line-up method, suggesting that human scents collected with our method are usable and valuable. In addition, the fact that no dogs ever committed FA during the last 100 trials confirmed the uniqueness of the human odours. Importantly, the matching-to-sample task was performed by the dogs without their handlers, and therefore the high Hit rate was solely attributable to the learning and olfactory abilities of the dogs and was not biased by any external influence of the handler [33, 34].

### Continuous training

Table 1 (S2 Data) presents results for the 10 periods of continuous training, each period comprising a mean 197.91 ± 2.15 trials. The total number of trials, Hits, Misses, FAs and CRs were summed per period per dog and used to calculate sensitivity (hits / [hits + misses]) and specificity (FAs / [FAs + CRs]). As shown in the Table, sensitivity significantly increased throughout continuous training, with scores ranging from 70.41 ± 2.56 in the 1^st^ period to 84.76 ± 3.83 in the 10^th^ period (F(9,83) = 2.47; *P* < 0.05). Specificity remained stable at the 100% ceiling in the last 4 periods (F(9,83) = 0.36; *P* n.s.). These results suggest that the sensitivity and specificity of human olfactory matching-to-sample improves with extensive training. Interestingly, all False Alarms were made by Belgian Shepherd dogs; the reason for this is unclear, however the time these dogs took to complete their line-up tasks suggests a decrease in level of attention.

**Table 1:** Proportion of correct detections (sensitivity scores: number of Hits / [Hits + Misses]) and False Alarms (specificity scores: number of CR / [CR + FA]) throughout the 10 periods of continuous training.

Periods	Scores of sensitivity ± S.E.M. (Hit / Hit + Miss) × 100	Scores of specificity ± S.E.M. (CR / CR + FA) × 100
1^st^	70.41 ± 2.56	100
2^nd^	73.46 ± 2.68	98.43 ± 1.22
3^rd^	75.50 ± 2.79	99.12 ± 0.88
4^th^	76.73 ± 2.92	100
5^th^	80.14 ± 3.72	99.00 ± 1.00
6^th^	82.62 ± 3.14	99.23 ± 0.77
7^th^	82.41 ± 4.19	100
8^th^	84.93 ± 2.69	100
9^th^	83.80 ± 4.99	100
10^th^	84.76 ± 3.83	100

**Fig 3:**
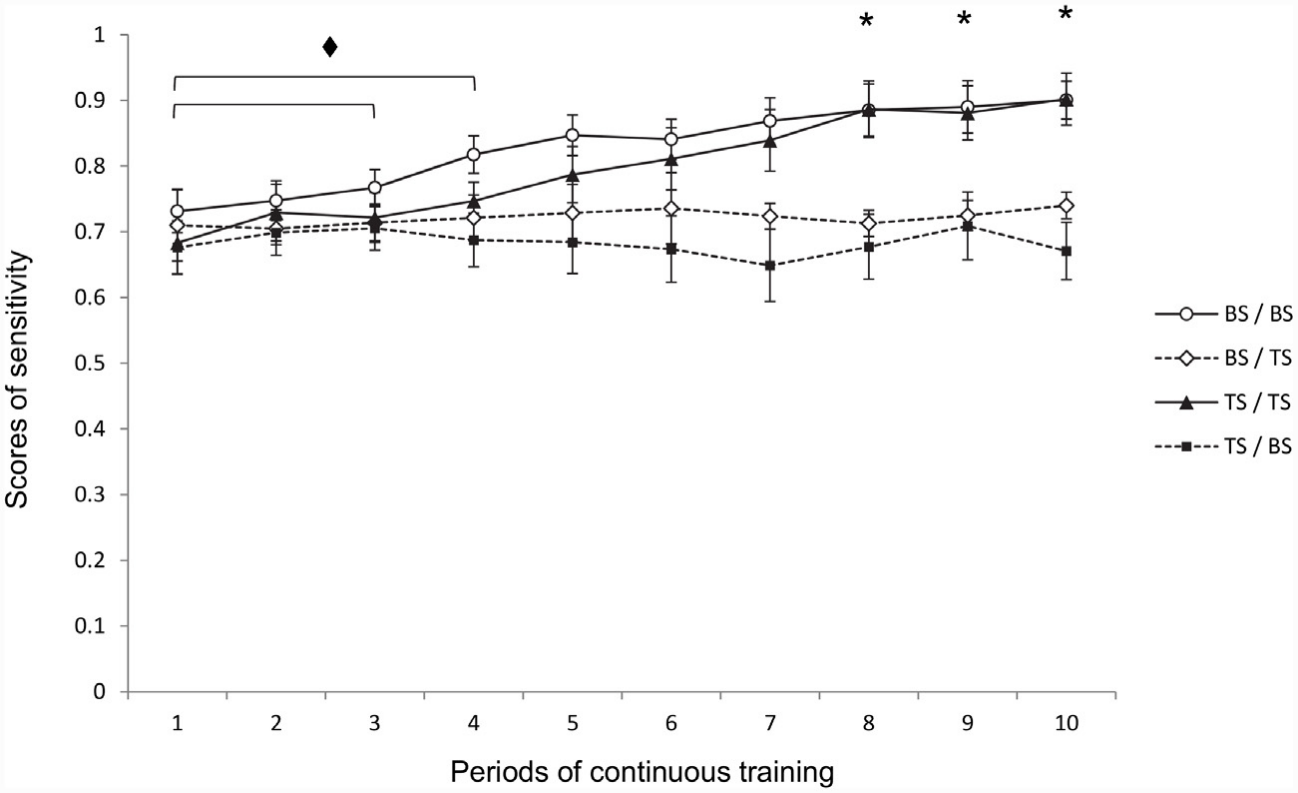
Effect of the type of scent combination used during continuous training on sensitivity scores. Sensitivity scores represent the proportion of correct detections (number of Hits / [Hits + Misses]). Each curve represents the evolution of scores (± S.E.M.) according to period. Intergroup comparison: * indicates *P* < 0.05 between BS / BS; TS / TS versus BS / TS and TS / BS in periods 8 to 10. Intragroup comparison: ♦indicates *P* < 0.05 compared to scores obtained in other periods for BS / BS and TS / TS.

Fig 3 (S2 Data) illustrates the sensitivity values (mean ± S.E.M.) for each combination of odour presentation in the 10 periods of continuous training. Sensitivity varied throughout continuous training depending on the type of odour combination. Two-factor ANOVA with repeated measures confirmed this, showing a significant effect of Type of Combination (F(3,16) = 3.33; *P* < 0.05) and of Period (F(9,144) = 3.77; *P* < 0.001) but without significant interaction between the two (F(27,144) = 0.76; *P* n.s.). Pairwise intergroup comparison (one-way ANOVA) indicated a significant effect of Type of Combination during the last 4 periods (F(3,28) = 4.81, *P* < 0.01; F(3,24) = 3.35, *P* < 0.05; F(3,20) = 3.77, P < 0.05; and F(3,16) = 6.50, *P* < 0.01, respectively). Within-group comparison showed a significant effect of Period for the BS/BS and TS/TS combinations but not for BS/TS or TS/BS (F(9,83) = 3.39, *P* < 0.001; and F(9,83) = 2.65, *P* < 0.01, respectively). These data suggest that sensitivity increased with the number of trials when the type of odour in the sample was similar to that presented in the line-up (BS/BS and TS/TS) but not when it differed (BS/TS and TS/BS).

### Court cases identification

Human odour identification in judicial cases took place from 2003 to 2014. The number of dogs that were assigned to the same judicial case has always ranged between 2 and 7 and the total number of line-ups per court case ranged between 13.83 ± 3.73 and 38.79 ± 3.07, and between 4.63 ± 0.44 and 12.99 ± 3.27 per dog, depending on the type of identification and the number of evidence scents. Hits and Misses were always confirmed with 100% consistency by the other dogs working on the same case (the same day or some days later, with the same scents but new jars). It is important to note that, as in previous reports [35], the success rates in identification were higher when the scent traces had been collected at the crime scene between within 24 hours of the offence (86.5% of cases; data not shown); when the interval was longer (13.5% of cases), the success rate decreased. Interestingly, confronting the suspect with a positive identification often leads to confession; since 2003, positive identifications made by dogs of the French DTSP in judicial cases helped to solve 120 criminal cases out of 435.

## Discussion

It is arguable that research on the forensic reliability of procedures based on dog scent capability has not adequately supported its widespread use in law enforcement. Regarding human scent identification by dogs, the question of the exact sensitivity and specificity of dogs’ line-up performance often arises.

The present study showed that our rigorous training procedure leads to a very high level of identification in human odour matching-to-sample tasks, with dogs alerting to target odours in 85 ± 4% of cases and never alerting to non-target human odours (100% specificity). According to Jezierski et al. (2014, [36]), high detection scores should be regarded as exceptional and indeed dubious as they depend on a variety of factors such as odour presentation method, odour source and, of course, individual differences in dogs’ olfactory detection thresholds. The present data confirm this attitude, inasmuch as sensitivity was critically dependent on the type of odour presentation during the task and specificity dependent on the dog’s breeding (FAs made exclusively by Belgian Shepherds).

The fact that the dogs’ ability to perform human odour line-ups was significantly higher when the type of odour in the sample and in the line-up was the same (BS/BS and TS/TS) clearly suggests that comparison between two odour samples of the same kind is much easier for dogs than comparison between two samples of different kinds. These two observations were also confirmed by the fact that all 8 False Alarms observed in the 18,127 trials in continuous training were obtained with the TS/BS combination and in Belgian Shepherds.

BS and TS likely consist of mixtures of various odorant compounds (body molecules + distractors) present in different proportions. Moreover, the proportion of distractors in the mixture affects the intensity of the targeted human odorants in the head space [37, 38] and interactions between odorant molecules in a mixture influence the detection and recognition of odorants in humans and in animals through activation of the olfactory sensory neurons in the nasal olfactory mucosa [39, 40]. If a Hit response depends directly on the degree of perceived similarity between body molecules present in the odour sample and those present in the line-up, then the difference in sensitivity suggests that the proportions of body molecules and distractors differ between BS and TS mixtures and that a common specific body scent feature is difficult for dogs to extract when both types of odour are used in the test. The excellent sensitivity scores obtained with BS/BS and TS/TS suggest the comparison could result from configural coding; however, the sensitivity scores obtained with BS/TS and TS/BS (ranging from 71% ± 3 to 74% ± 3; data not shown) suggest that dogs were able to extract common specific body scent information from mixtures presented in the sample and in the line-up by an elemental coding process.

Interestingly, our results show an increase in sensitivity during training, suggesting that the ability of dogs to perform human matching-to-sample can improve with the number of trials. Therefore, future studies will focus on increasing hit rates with BS/TS and TS/BS combinations in order to enhance reliability and the number of Hits in judicial identification tasks. The fact that FAs were committed exclusively by Belgian Shepherds and that sensitivity was higher in German Shepherds suggests that the latter breed should be preferred in future procedures.

## Conclusion

Despite dogs’ demonstrated ability to discriminate and identify human scent with the line-up technique, the admissibility of such evidence is not systematically accepted by the forensic community and the courts and is often challenged in some countries. The present study shows that rigorous procedures and continuous training lead to high sensitivity and specificity on human olfactory matching-to-sample tasks. The high reproducibility of the scores during continuous training guarantees the accuracy of results in judicial identification tests. Given that positive identification merely establishes a direct or indirect relationship between suspect and crime scene, the information gained from the human scent identification line-up technique by certified dogs should, if used with discretion, provide a valuable tool for law enforcement and should be used in court as “additional forensic evidence”.

## Supporting Information

S1 Data Summary of the data (mean S.E.M.) obtained during successive phases of intitial learning. These data refer to Fig 2.
(XLSX)

S2 Data Summary of the data (mean S.E.M.) obtained during the 10 periods of continuous training. These data refer to Fig 3 and Table 1.
(XLSX)
